# Jinlida granules combined with metformin improved the standard-reaching rate of blood glucose and clinical symptoms of patients with type 2 diabetes: secondary analysis of a randomized controlled trial

**DOI:** 10.3389/fendo.2023.1142327

**Published:** 2023-05-25

**Authors:** Xiaomin Kang, Yuting Sun, Yingying Duan, Yuqing Zhang, Xudong An, De Jin, Fengmei Lian, Xiaolin Tong

**Affiliations:** ^1^ Guang’anmen Hospital, China Academy of Chinese Medical Sciences, Beijing, China; ^2^ Graduate School, Beijing University of Chinese Medicine, Beijing, China; ^3^ Department of Nephrology, Hangzhou Hospital of Traditional Chinese Medicine, Hangzhou, China

**Keywords:** Jinlida, Chinese medicine, type 2 diabetes, clinical symptom, secondary analysis

## Abstract

**Background:**

Previous studies found that Jinlida granules could significantly reduce blood glucose levels and enhance the low-glucose action of metformin. However, the role of Jinlida in the standard-reaching rate of blood glucose and improving clinical symptoms has yet to be studied. We aimed to elaborate on the efficacy of Jinlida in type 2 diabetes (T2D) patients who experience clinical symptoms based on secondary analysis of a randomized controlled trial.

**Methods:**

Data were analyzed from a 12-week, randomized, placebo-controlled study of Jinlida. The standard-reaching rate of blood glucose, the symptom disappearance rate, the symptom improvement rate, the efficacy of single symptoms, and the total symptom score were evaluated. The correlation between HbA1c and the improvement of clinical symptoms was analyzed.

**Results:**

For 12 weeks straight, 192 T2D patients were randomly assigned to receive either Jinlida or a placebo. The treatment group showed statistically significant differences in the standard-reaching rate of HbA1c < 6.5% (*p* = 0.046) and 2hPG (< 10 mmol/L, 11.1 mmol/L) (*p* < 0.001), compared with the control group. The standard-reaching rate of HbA1c < 7% (*p* = 0.06) and FBG < 7.0 mmol/L (*p* = 0.079) were not significantly different between the treatment and control groups. Five symptoms exhibited a statistical difference in symptom disappearance rate (*p* < 0.05). All the symptoms exhibited a significant difference in symptom improvement rate (*p* < 0.05). The mean change in total symptom score from baseline to week 12 was −5.45 ± 3.98 in the treatment group and −2.38 ± 3.11 in the control group, with statistically significant differences (*p* < 0.001). No significant correlations were noted between symptom improvement and HbA1c after 12 weeks of continuous intervention with Jinlida granules or placebo.

**Conclusion:**

Jinlida granules can effectively improve the standard-reaching rate of blood glucose and clinical symptoms of T2D patients, including thirst, fatigue, increased eating with rapid hungering, polyuria, dry mouth, spontaneous sweating, night sweat, vexing heat in the chest, palms, and soles, and constipation. Jinlida granules can be used as an effective adjuvant treatment for T2D patients who experience those symptoms.

## 1 Introduction

Diabetes is becoming an increasingly serious global public health issue. The International Diabetes Federation (IDF) shows that patients with diabetes mellitus are on the rapid rise worldwide, and the number of patients with diabetes worldwide is expected to reach 783 million by 2045 (1). An epidemiological investigation exhibited that the estimated prevalence of diabetes in China in 2018 was 12.4%, and only 50.1% of treated diabetic patients were adequately controlled (2). People living with diabetes are at risk of developing several serious and life-threatening complications, leading to an increased need for medical care, reduced quality of life, and undue stress on families (3). Diabetes and its complications, if not well managed, can lead to frequent hospital admissions and premature death (4). Despite the emergence of new antidiabetic drugs, the glycemic control achieved is far from perfect, and some drugs may cause adverse events and increase cardiovascular disease as well as ineffectively improve the clinical symptoms (5–8).

Therefore, there is an urgent need for new drugs that are effective with minimal side effects and favorable hypoglycemic effects, as well as relieving clinical symptoms. Traditional Chinese medicine (TCM) has been applied in the treatment of diabetes for thousands of years. In recent years, TCM has made significant progress in modernization and globalization. Jinlida granule is a Chinese patent medicine composed of 17 Chinese herbs (Ginseng, rhizoma polygonati, rhizoma atractylodis lanceae, sophorae flavescentis, ophiopogon japonicus, rehmanniae, polygoni multiflori, dogwood, poria perrin, eupatorium, coptis chinensis, anemarrhena, epimedium, salvia, puerariae, semen litchi, and cortex lycii radices). It was approved by the China Food and Drug Administration (CFDA) as a treatment drug for type 2 diabetes (T2D) in 2005 (National Drug Approval No. Z20050845) and has been widely used in clinical practice (9). In the previous work, our research team conducted a randomized, placebo-controlled clinical trial to evaluate the efficacy and safety of Jinlida in T2D with a sample size of 192 subjects who received the Jinlida or placebo for 12 weeks based on using metformin. Our results showed that glycosylated hemoglobin (HbA1c) was reduced more significantly in the Jinlida group (10, 11). Furthermore, meta-analysis and systematic reviews of Jinlida granules showed significant reductions in fasting blood glucose (FBG), 2-h postprandial blood glucose (2hPG), and HbA1c (12, 13). Animal experiments show that Jinlida granules could promote the thermogenesis of brown and beige adipocytes by enhancing the mitochondrial function and inhibiting the expression of miR-27a, thereby improving the metabolism of glucose and lipids (14, 15). Jinlida granules could also reduce insulin resistance in rats fed with high fat by regulating phosphorylation of c-Jun N-terminal kinase (JNK) and p38 mitogen-activated protein kinase (MAPK) (16). However, as previously reported, current studies in Jinlida put more emphasis on the effect of blood glucose levels and their potential mechanisms and largely ignore the role of improving clinical symptoms in T2D.

Though the efficacy and safety of Jinlida, as well as its potential action mechanisms, were elucidated, the role of improving clinical symptoms in Jinlida has yet to be studied. Hence, in this work, we conducted a secondary analysis based on the previous randomized controlled trial (10) to investigate the role of the standard-reaching rate of blood glucose and improving clinical symptoms in Jinlida to bridge the gap between hypoglycemic agents and improvement in diabetic clinical symptoms.

## 2 Methods

### 2.1 Study design and population

A 12-week randomized controlled trial was designed to evaluate the efficacy of Jinlida as an add-on medication in T2D. All subjects in both groups also continuously received their metformin without any dose changes. Treatment group: Jinlida granules were orally given at 9 g three times per day after meals. Control group: placebos were orally given at 9 g three times per day after meals. The eligible subjects were T2D patients who received metformin treatment for more than 3 months but HbA1c ≥ 7.0% and FPG 7.0–13.9 mmol/L or 2hPG ≥ 11.1 mmol/L. Patients who took other hypoglycemic drugs or insulin besides metformin in the past 3 months were excluded. People with complications of diabetes, severe kidney, liver, or cardiovascular disease, or mental illness, and who were pregnant or preparing to become pregnant were also excluded.

### 2.2 Outcomes

The primary outcomes were the standard-reaching rates of blood glucose, including HbA1c (HbA1c < 6.5%, < 7%), FBG (< 7 mmol/L), and 2hPG (< 10mmol/L, 11.1 mmol/L) in the two groups after 12 weeks of treatment. The secondary outcomes were symptom disappearance rate, symptom improvement rate, efficacy of single symptom, and total symptom score. During this 12-week study period, all the subjects received a clinical symptom evaluation at 0, 4, 8, and 12 weeks. The assessed symptoms were nine common symptoms of patients with T2D, including thirst, fatigue, increased eating with rapid hungering, polyuria, dry mouth, spontaneous sweating, night sweat, vexing heat in the chest, palms, and soles, and constipation. The efficacy of a single symptom was marked as disappearance, improvement, and ineffective. It was scored according to the severity of symptoms. None, mild, moderate, and severe of the thirst and fatigue symptoms were scored 0, 2, 4, and 6, respectively, and of the other seven symptoms were scored 0, 1, 2, and 3, respectively. The cumulative score of the nine symptoms was the total symptom score. A correlation analysis between symptom improvement (change of the total symptom score) and HbA1c was also evaluated.

### 2.3 Statistics analysis

The primary analyses were performed according to the intention-to-treat (ITT) principle. The missing data were processed by the last observation carried forward (LOCF). The counting data were described by composition ratio. The measurement data are described by mean ± standard deviation. Group *t*-tests or Wilcoxon rank sum tests were used to compare measurement data between groups. A paired *t*-test was used to compare the changes in symptom scores before and after the intervention. The classification variables were compared using the Chi-square test. The level of statistical significance was set at *p* < 0.05. SPSS 19.0 software (SPSS Inc., Chicago, IL, USA) was used to analyze all the data. The correlation between symptom improvement and HbA1c was analyzed through Pearson correlation analysis.

## 3 Results

### 3.1 Patients

In this trial, 186 participants completed the study, including 94 in the placebo group and 92 in the Jinlida group. There was no statistically significant difference in HbA1c, FBG, 2hPG, body weight, and BMI between Jinlida and placebo groups at baseline. Demographic information of subjects has been reported previously (10). Symptom assessment of subjects at baseline is shown in **Table 1**.

**Table 1:** The symptom assessment of subjects in the treatment and control groups at baseline.

Index	Treatment group	Control group		Rank sum test			
				*Z*-value		*p*-value	
Thirst							
0 score (*n* (%))	12 (13.0)	14 (14.9)		−0.465		0.642	
2 score (*n* (%))	50 (54.3)	50 (53.2)					
4 score (*n* (%))	22 (23.9)	27 (28.7)					
6 score (*n* (%))	8 (8.7)	3 (3.2)					
Total (*n* (%))	92(100.0)	94(100.0)					
Fatigue							
0 score (*n* (%))	17 (18.5)	8 (8.5)		−1.807		0.071	
2 score (*n* (%))	61 (66.3)	67 (71.3)					
4 score (*n* (%))	14 (15.2)	19 (20.2)					
6 score (*n* (%))	0 (0.0)	0 (0.0)					
Total (*n* (%))	92 (100.0)	94 (100.0)					
Increased eating with rapid hungering							
0 score (*n* (%))	22 (23.9)	19 (20.2)		−0.376		0.707	
1 score (*n* (%))	47 (51.1)	51 (54.3)					
2 score (*n* (%))	22 (23.9)	24 (25.5)					
3 score (*n* (%))	1 (1.1)	0 (0.0)					
Total (*n* (%))	92 (100.0)	94 (100.0)					
Polyuria							
0 score (*n* (%))	20 (21.7)	17 (18.1)		−0.095		0.924	
1 score (*n* (%))	44 (47.8)	51 (54.3)					
2 score (*n* (%))	26 (28.3)	23 (24.5)					
3 score (*n* (%))	2 (2.2)	3 (3.2)					
Total (*n* (%))	92 (100.0)	94 (100.0)					
Dry mouth							
0 score (*n* (%))	12 (13.0)	23 (24.5)		−1.526		0.127	
1 score (*n* (%))	42 (45.7)	38 (40.4)					
2 score (*n* (%))	37 (40.2)	32 (34.0)					
3 score (*n* (%))	1 (1.1)	1 (1.1)					
Total (*n* (%))	92 (100.0)	94 (100.0)					
Spontaneous sweating							
0 score (*n* (%))	29 (31.5)	35 (37.2)		−0.617		0.537	
1 score (*n* (%))	51 (55.4)	46 (48.9)					
2 score (*n* (%))	10 (10.9)	13 (13.8)					
3 score (*n* (%))	2 (2.2)	0 (0.0)					
Total (*n* (%))	92 (100.0)	94 (100.0)					
Night sweat							
0 score (*n* (%))	52 (56.5)	52 (55.3)		−0.123		0.902	
1 score (*n* (%))	32 (34.8)	34 (36.2)					
2 score (*n* (%))	6 (6.5)	7 (7.4)					
3 score (*n* (%))	2 (2.2)	1 (1.1)					
Total (*n* (%))	92(100.0)	94(100.0)					
Vexing heat in the chest, palms, and soles							
0 score (*n* (%))	29 (31.5)	38 (40.4)		−0.675		0.500	
1 score (*n* (%))	49 (53.3)	38 (40.4)					
2 score (*n* (%))	12 (13.0)	18 (19.1)					
3 score (*n* (%))	2 (2.2)	0 (0.0)					
Total (*n* (%))	92 (100.0)	94 (100.0)					
Constipation							
0 score (*n* (%))	36 (39.1)	38 (40.4)		−0.255		0.798	
1 score (*n* (%))	47 (51.1)	48 (51.1)					
2 score (*n* (%))	9 (9.8)	8 (8.5)					
3 score (*n* (%))	0 (0.0)	0 (0.0)					
Total (*n* (%))	92 (100.0)	94 (100.0)					

### 3.2 Standard-reaching rate of blood glucose

The standard-reaching rates of HbA1c (< 6.5%, < 7%) were 25.0% (16.6%~35.1%) and 43.5% (33.2%~54.2%) in the treatment group, compared with 14.9% (8.4%~23.7%) and 31.9% (22.7%~42.3%) in the control group. The standard-reaching rate of FBG (< 7 mmol/L) was 34.8% (25.2%~45.4%) in the treatment group and 23.4% (15.3%~33.3%) in the control group. The standard-reaching rates of 2hPG (< 10 mmol/L, < 11.1 mmol/L) were 32.6% (23.2%~43.2%) and 39.1% (29.1%~49.9%) in the treatment group, compared with 10.6% (5.2%~18.7%) and 18.1% (10.9%~27.4%) in the control group. The treatment group showed statistically significant differences in the standard-reaching rate of HbA1c < 6.5% (*p* = 0.046), and 2hPG (< 10 mmol/L, 11.1 mmol/L) (*p* < 0.001), compared with the control group. Although the standard-reaching rates of HbA1c < 7% (*p* = 0.06) and FBG < 7.0 mmol/L (*p* = 0.079) were not significantly different between the treatment and control groups, the trend was significant (**Table 2**).

**Table 2:** The standard-reaching rates of blood glucose in the treatment and control groups after 12 weeks of intervention.

Standard-reaching outcomes	Treatment group	Control group	*p*-value
HbA1c < 6.5%	25.0% (16.6%~35.1%)	14.9% (8.4%~23.7%)	0.046
HbA1c < 7.0%	43.5% (33.2%~54.2%)	31.9% (22.7%~42.3%)	0.06
FBG < 7.0 mmol/L	34.8% (25.2%~45.4%)	23.4% (15.3%~33.3%)	0.079
2hPG < 10.0 mmol/L	32.6% (23.2%~43.2%)	10.6% (5.2%~18.7%)	0.000
2hPG < 11.1 mmol/L	39.1% (29.1%~49.9%)	18.1% (10.9%~27.4%)	0.000

The 95% confidence interval of the standard-reaching rates of the two groups was estimated using the accurate method (Clopper–Pearson).

### 3.3 Symptom disappearance rate

**Figure 1:**
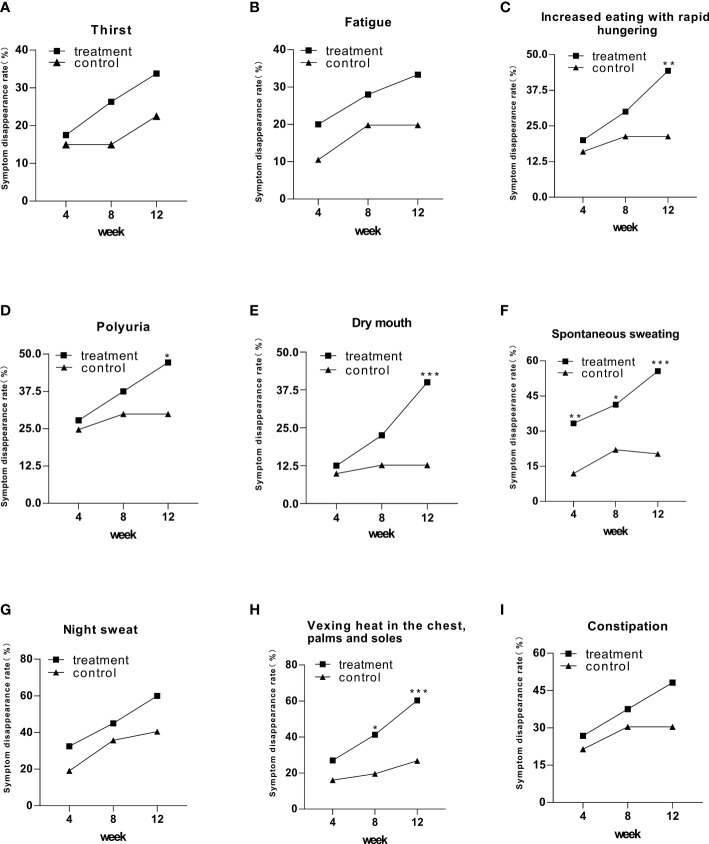
The symptom disappearance rate in the treatment and control groups after 12 weeks of intervention. Comparison of outcomes between groups: ^*^ *p* < 0.05; ^**^ *p* < 0.01; ^***^ *p* < 0.001.

All the symptom disappearance rates had an upward trend (**Figure 1**). However, only five of nine symptoms exhibited a statistical difference in the treatment group, including increased eating with rapid hungering, polyuria, dry mouth, spontaneous sweating, and vexing heat in the chest, palms, and soles, compared to that in the control group (**Figure 1**). Especially in spontaneous sweating, compared with the control group, the treatment group showed a significant difference at week 4, with a faster rate of clinical improvement than other symptoms (**Figure 1**).

### 3.4 Symptom improvement rate

**Figure 2:**
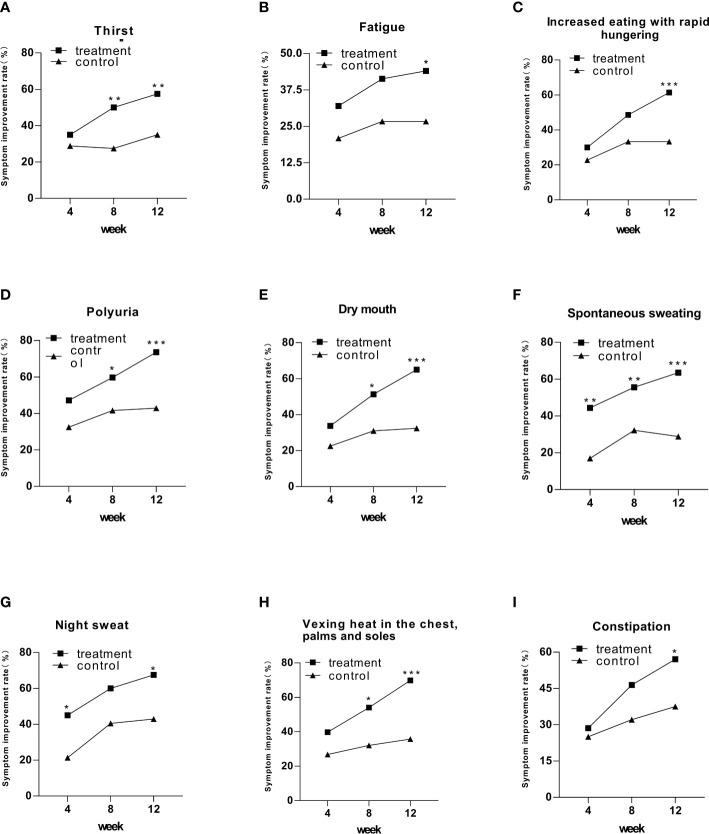
The symptom improvement rate in the treatment and control groups after 12 weeks of intervention. Comparison of outcomes between groups: ^*^ *p* < 0.05; ^**^ *p* < 0.01; ^***^ *p* < 0.001.

After 12 weeks of intervention, all symptoms showed statistically significant differences in symptom improvement rates in the treatment group compared to the control group (**Figure 2**). Four of nine symptoms exhibited a significant difference in the treatment group at week 8, including thirst, polyuria, dry mouth, and vexing heat in the chest, palms, and soles, compared to that in the control group (**Figure 2**). Spontaneous sweating symptoms in the treatment group achieved a statistically significant difference at week 4 (**Figure 2**). Notably, though night sweat symptoms achieved a statistically significant difference in weeks 4 and 12, they did not exhibit a significant difference in week 8 (**Figure 2**).

### 3.5 The efficiency of a single symptom

**Figure 3:**
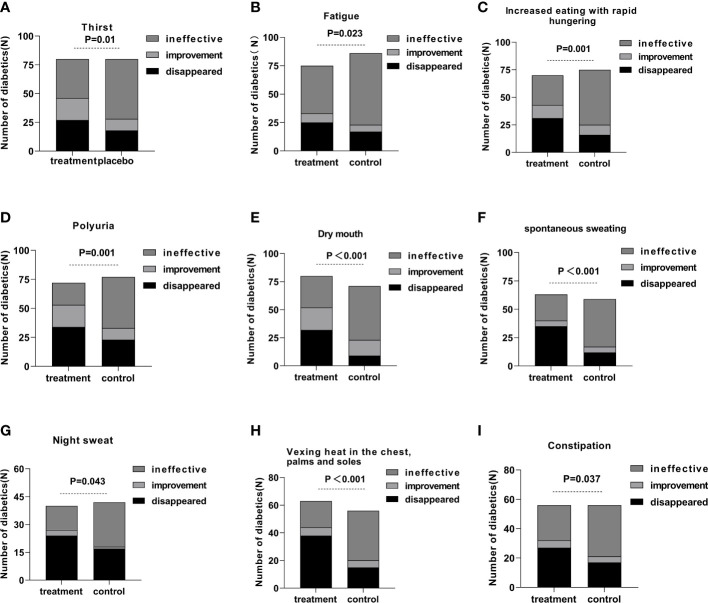
The efficiency of a single symptom in the treatment and control groups after 12 weeks of intervention.

Every symptom was evaluated as ineffective, improved, and disappeared. All the symptoms exhibited a statistical difference comparing the treatment group to the control group after 12 weeks of continuous intervention (**Figure 3**).

### 3.6 Total symptom score

**Figure 4:**
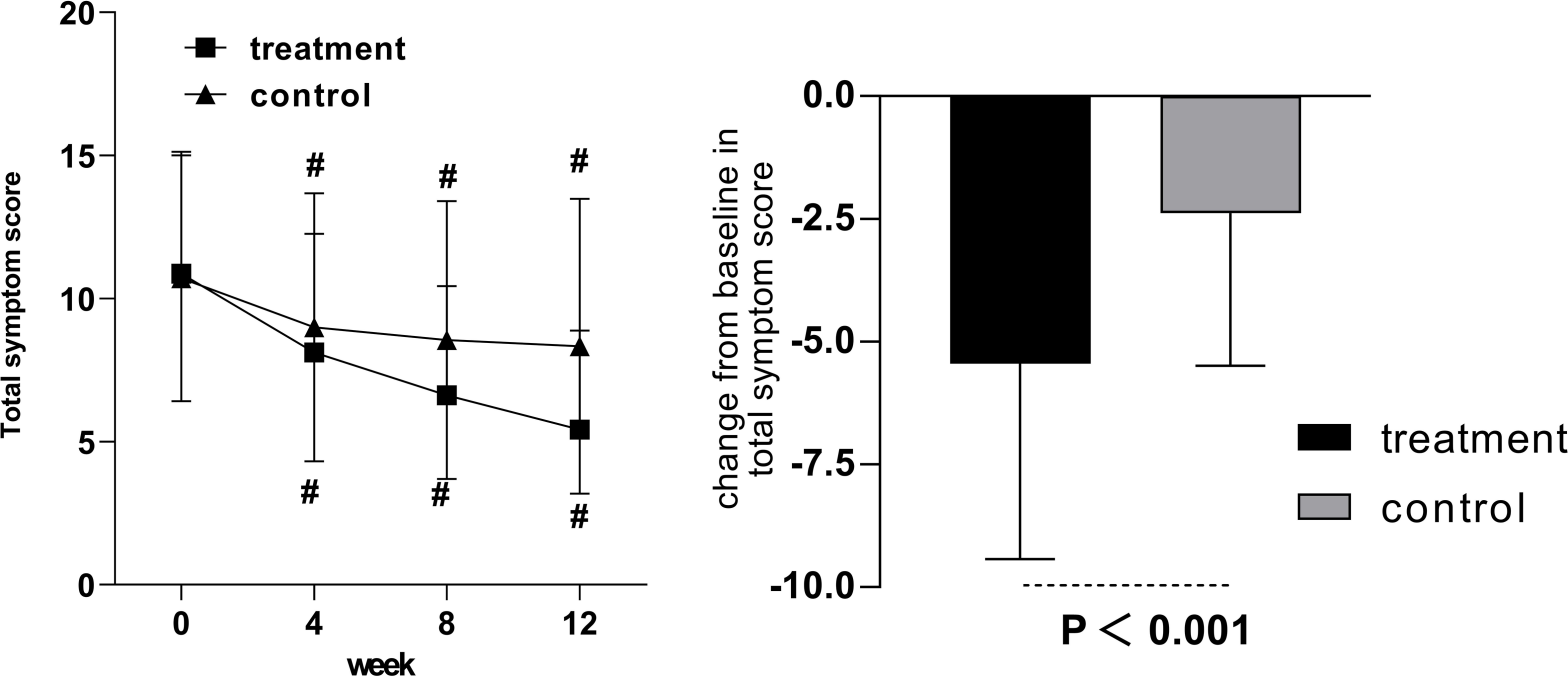
The total symptom score in the treatment and control groups after 12 weeks of intervention. Comparison of outcomes with baseline: ^#^ *p* < 0.001.

Compared with pretreatment, the total symptom score was statistically significant in both groups in weeks 4, 8, and 12 (**Figure 4**). The mean change in total symptom score from baseline to week 12 was −5.45 ± 3.98 in the treatment group and −2.38 ± 3.11 in the control group, with statistically significant differences.

### 3.7 Correlation of symptom improvement and HbA1c

**Figure 5:**
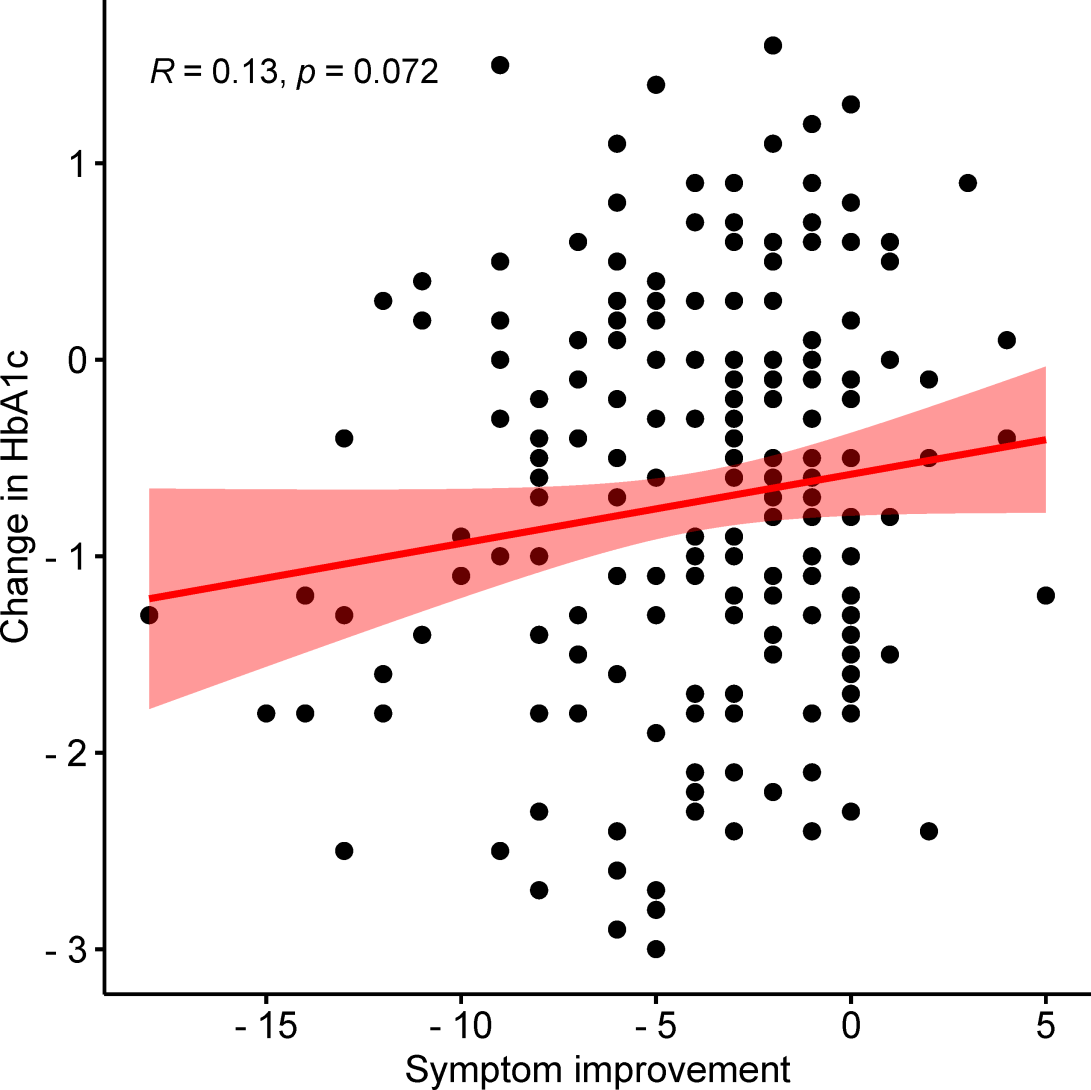
The correlation between HbA1c and the improvement of clinical symptoms.

Pearson correlation was applied to analyze the correlation between symptom improvement and HbA1c. The correlation coefficient was 0.13, and the *p*-value was 0.072. No significant correlations were noted between symptom improvement and HbA1c after 12 weeks of continuous intervention with Jinlida granules or placebo (**Figure 5**).

## 4 Discussion

The control of T2D has become a global challenge. For the management of diabetes, there are various oral hypoglycemic drugs (OHDs) that are available worldwide, which is major management for most T2D patients. Of all the OHAs to treat T2D, metformin is the first-line oral hypoglycemic agent for the treatment of T2D recommended by the American Diabetes Association (17). However, like many lowering-glucose agents, metformin may not improve clinical symptoms despite its favorable lowering-glucose effects. The treatment strategy for T2D patients is inadequate. The necessity to develop new strategies for diabetic patients is in high demand. Clinical studies, including randomized double-blind placebo-controlled trials, have shown that the use of traditional Chinese medicine can significantly reduce HbA1c levels and improve clinical symptoms in patients with T2D (18, 19).

In TCM theory, the pathogenesis of T2D is closely related to Pi (spleen) deficiency. Pi (spleen) deficiency usually leads to clinical symptoms such as fatigue, thirst, and abnormal sweating. Jinlida granules is a herbal formula developed according to the Chinese understanding of the pathogenesis theory of diabetes, Pi (spleen) deficiency. It reduces blood glucose and improves clinical symptoms by nourishing Pi (spleen) and regulating the body fluid of diabetic patients (20, 21).

From the current evidence of the trials, both Jinlida and metformin reduced the level of blood glucose, and the curative effect of Jinlida add-on metformin was superior to metformin monotherapy (10–13). In terms of the standard-reaching rate of blood glucose, Jinlida add-on metformin has obvious advantages in comparison to metformin monotherapy. Jinlida add-on metformin could improve the standard-reaching rate of blood glucose. Notably, HbA1c < 7% and FBG < 7mmol/L did not show a statistically significant difference but exhibited a standard-reaching tendency. The nonsignificant finding might be related to the small sample size.

In the aspect of improving clinical symptoms, Jinlida granules have an improvement effect on all symptoms, especially the five symptoms: increased eating with rapid hunger, polyuria, dry mouth, spontaneous sweating, and vexing heat in the chest, palms, and soles. Among them, the disappearance rate of spontaneous sweating symptoms was statistically significant after 4 weeks of treatment. It shows that spontaneous sweating is the most effective symptom of Jinlida granules. At weeks 4 and 12, the night sweat symptom in the treatment and control groups showed significant differences. At week 8, the night sweat symptom showed an insignificant difference. The curative effect was improved in a spiral pattern, indicating that the effect of Jinlida granules was mild and stable. In addition, we analyzed the correlation between HbA1c and the improvement of clinical symptoms, and there was no significant difference. Therefore, the effect of Jinlida on improving clinical symptoms is considered to be independent of regulating blood glucose.

There were also some limitations in the present study. First, subjectivity in assessing subjective symptoms is a notable confounding factor. Second, due to the long follow-up interval, the patient’s symptoms may not be able to get timely feedback. Third, the 12-week intervention period is short, and the long-term benefits of Jinlida granules in improving clinical symptoms are unclear. Future research should focus on developing more effective strategies to evaluate subjective clinical symptoms and longer follow-up times to evaluate the improvement of T2D clinical symptoms.

## 5 Conclusion

Jinlida granules can effectively improve the standard-reaching rate of blood glucose and clinical symptoms of T2D patients, including thirst, fatigue, increased eating with rapid hungering, polyuria, dry mouth, spontaneous sweating, night sweat, vexing heat in the chest, palms, and soles, and constipation, and their action might be independent of regulating blood glucose. Jinlida granules can be used as an effective adjuvant treatment for T2D patients who experience those symptoms. However, these findings need to be further confirmed by evidence-based medicine (22).

## Data availability statement

The raw data supporting the conclusions of this article will be made available by the authors, without undue reservation.

## Ethics statement

The studies involving human participants were reviewed and approved by the Guang’anmen Hospital Medical Ethics Commission in China. The patients/participants provided their written informed consent to participate in this study.

## Author contributions

XK and YS wrote the manuscript. YD, YZ, and XA performed the statistical analysis. DJ, FL, and XT designed the protocol and revised the paper. All authors contributed to the article and approved the submitted version.
